# *In vitro* and *in vivo* anti−*Toxoplasma* activities of HDAC inhibitor Panobinostat on experimental acute ocular toxoplasmosis

**DOI:** 10.3389/fcimb.2022.1002817

**Published:** 2022-09-12

**Authors:** Yu Zhang, Qingqing Zhang, Haiming Li, Hua Cong, Yi Qu

**Affiliations:** ^1^ Department of Geriatrics, Qilu Hospital of Shandong University, Jinan, China; ^2^ Department of Pathogen Biology, School of Basic Medical Sciences, Cheeloo College of Medicine, Shandong University, Jinan, China

**Keywords:** ocular toxoplasmosis, HDAC inhibitor, anti-*Toxoplasma gondii*, therapy, ocular inflammation, intravitreal injection

## Abstract

Ocular toxoplasmosis (OT) is retinochoroiditis caused by *Toxoplasma gondii* infection, which poses a huge threat to vision. However, most traditional oral drugs for this disease have multiple side effects and have difficulty crossing the blood-retinal barrier, so the new alternative strategy is required to be developed urgently. Histone deacetylases (HDAC) inhibitors, initially applied to cancer, have attracted considerable attention as potential anti-*Toxoplasma gondii* drugs. Here, the efficacy of a novel HDAC inhibitor, Panobinostat (LBH589), against *T. gondii* has been investigated. *In vitro*, LBH589 inhibited the proliferation and activity of *T. gondii* in a dose-dependent manner with low toxicity to retinal pigment epithelial (RPE) cells. *In vivo*, optical coherence tomography (OCT) examination and histopathological studies showed that the inflammatory cell infiltration and the damage to retinal architecture were drastically reduced in C57BL/6 mice upon treatment with intravitreal injection of LBH589. Furthermore, we have found the mRNA expression levels of inflammatory cytokines were significantly decreased in LBH589–treated group. Collectively, our study demonstrates that LBH589 holds great promise as a preclinical candidate for control and cure of ocular toxoplasmosis.

## Introduction

*Toxoplasma gondii*, a protozoan of the Apicomplexa Phylum, is a highly infectious obligate intracellular parasite (Khosravi et al., 2020). Ocular toxoplasmosis is an inflammatory disease caused by intraocular infection with *T. gondii* (Garweg, 2016). When tachyzoites cross the blood retinal-barrier and access to the retina, they may infect any nucleated host cells, causing tissue damage and visual impairment (Jones et al., 2017). The typical manifestation of acute disease is usually unilateral necrotizing retinochoroiditis accompanied by severe vitritis (Patel and Vavvas, 2022). In addition, other ocular atypical manifestations and complications include anterior uveitis, cataract, retinal neuritis, scleritis and retinal detachment (Kalogeropoulos et al., 2022). Toxoplasmosis has been proposed to be a leading cause of retinal infection, accounting for 20-60% of the total cases of posterior uveitis worldwide (Fabiani et al., 2022). The incidence rate of OT is about 2% in clinically diagnosed cases of *T. gondii* infection (Kijlstra and Petersen, 2014).

Currently, the clinical treatment of *T. gondii* infection is generally a systematic combination of several oral antimicrobials that target the parasite such as pyrimethamine and sulfadiazine (Feliciano-Alfonso et al., 2021). However, many patients with ocular toxoplasmosis manifest with ocular symptoms alone but systemic symptoms are not obvious. The current problem caused by traditional medication lies in the side effects such as anemia and visceral toxicity; on the other hand, it is difficult for most drugs to cross the blood-retinal barrier to reach effective drug concentration in the eye, leading to the result: the disease cannot be quickly controlled in the acute phase of the disease (Garweg and Pleyer, 2021). To overcome this predicament, there is a recognized necessity to develop new anti-*T. gondii* drugs and treatment strategies.

In recent years, HDAC inhibitors have been developed as an attractive class of targeted agents against cancers in recent years (McClure et al., 2018). However, these compounds have been revealed to induce hyperacetylation in histone and non-histone proteins in tumor cells, causing cell cycle arrest, senescence and apoptosis, and have promising clinical outcomes in hematological neoplasms (Bondarev et al., 2021). Notably, the latest research suggests HDAC inhibitors have been proposed as a potential alternative agent for protozoan infection such as *T. gondii* (Engel et al., 2015; Araujo-Silva et al., 2021). It has been demonstrated that these compounds are resistant to *T. gondii* mainly by controlling the acetylation status of histones, affecting the life cycle of *T. gondii* and leading to the destruction of the microstructure (Mouveaux et al., 2022; Jublot et al., 2022). However, there are relatively few evaluations of the therapeutic effect of HDAC inhibitors on local target organ infections such as OT.

In this study, we have investigated a novel HDAC inhibitor, LBH589, against the infection of *T. gondii*. *In vitro*, RPE cells were selected as host cells to evaluate the inhibitory effect of LBH589 on *T. gondii*. Notably, we have ascertained the LBH589’s ability to control this disease by intravitreal injection, using a mouse model of acute OT. We hope that this study could provide new ideas for the treatment of OT.

## Materials and methods

### Parasites and cell culture

*T. gondii* tachyzoites of RH and RH-GFP strains were cultured and passaged in human foreskin fibroblast (HFF) cells. Tachyzoites were purified and quantified prior to infecting the cells and mice. Human retinal pigment epithelial cells (ARPE-19, FuHeng Biology, Shanghai, China) were serially cultured in 25 cm culture flasks, and the first 30 passages were used for experiments. All cells above were grown in Dulbecco’s modified Eagle’s medium (DMEM; Gibco™, USA) supplemented with 10% inactivated fetal bovine serum (FBS; Gibco™, Australia) and 1% penicillin-streptomycin (Solarbio, Beijing, China) at 37°C in sterile atmosphere containing 5% CO_2_.

### Histone deacetylases inhibitor

LBH589 (Selleck, Shanghai, China) was dissolved in dimethyl sulfoxide (DMSO; Solarbio, Beijing, China), and diluted subsequently in DMEM or phosphate-buffered saline (PBS; Meilunbio, Dalian, China) to different working concentrations for use. To prevent the toxic effects, the final concentration of DMSO should not exceed 0.01% in all experiments.

### Cytotoxicity assay

RPE cells (5 × 10^3^ cells/well) were seeded in 96-well plates and grown for 24 h. The medium containing different concentrations of LBH589 (10, 5, 2.5, 1.0, 0.75, 0.5, 0.25 μM) was changed. After the cells were treated with drugs for 48 h, 10 μl Cell Counting Kit-8 reagent (CCK-8; meilunbio, Dalian, China) was added to each well and incubated for 1 h at 37°C in a dark environment. Absorbance was measured at 450 nm by a microplate reader (Tecan, Nanjing, China) and statistical analysis was performed with Graphpad Prism 8 to calculate the 50% cytotoxic concentrations (CC50).

### Antiproliferative assay

RPE cells were seeded in 24-well plate until a cell monolayer formed, and RH-GFP tachyzoites were added to each well at a ratio of 10:1 to cells. After 4 h of infection, drug-containing medium (750, 500, 250 nM) was replaced to each well and continued to maintain for 48 h. The proliferation of tachyzoites was observed by randomly selecting fields under the inverted fluorescence microscope (ZEISS, Germany). In order to better observe the intracellular proliferation of parasite, the cells were infected with RH tachyzoites and treated with the drug (750, 375 nM) for 48 h. Staining according to Wright-Giemsa Stain solution instruction (Solarbio, Beijing, China), random fields of view were selected under light microscope for observation. The image data obtained in the experiment were analyzed with image-J.

### Plaque assay

Free RH tachyzoites were purified and pretreated with LBH589 (750, 375 nM) for 8 h, then confluent monolayers of RPE cells in 12-well plate were infected with tachyzoites (500/well) for 10 days. The plate was stained according to the instructions of Crystal Violet Staining Solution (Beyotime, Shanghai, China) and visualized under microscope. Statistical analysis of the number and size of all plaques was performed with image-J.

### mRNA expression analysis by using quantitative real-time reverse transcription-polymerase chain reaction (qRT-PCR)

Total RNA was extracted from mouse eyes and cell lines using an RNAfast 200 total RNA rapid extraction kit (Feijie Biotechnology, Shanghai, China). After the quantity and purity were determined by NanoDrop 2000 (Thermo, Shanghai, China), 1 μg of total RNA was used to generate cDNA by miDETECT A Track miRNA qRT-PCR Starter Kit (RiboBio, Guangzhou, China). Quantitative RT-PCR analysis was performed using Blaze Taq™ SYBR Green qPCR Mix 2.0 (GeneCopoeia, Guangzhou, China) to detect the mRNA levels of interleukin (IL)-1β, IL-6, IL-8, granulocyte-macrophage colony-stimulating factor (GM-CSF), and tumor necrosis factor (TNF)-α. Primers used for qRT-PCR are listed in **Table 1**. The relative mRNA expression of each target gene was normalized to that of the housekeeping gene β-actin and GAPDH, and the result was fold-changed compared to the blank control group (set to 1).

**Table 1:** The primer Sequences of genes.

Gene	Sequences (5’-3’)	
	Forward primer	Reverse primer
Mus-IL-1β	GTGTCTTTCCCGTGGACCTT	AATGGGAACGTCACACACCA
Mus-IL-6	CTTCTTGGGACTGATGCTGGT	CTCTGTGAAGTCTCCTCTCCG
Mus-TNF-α	AGCCGATGGGTTGTACCTTG	ATAGCAAATCGGCTGACGGT
Mus-GAPDH	TGTCTCCTGCGACTTCAACA	GGTGGTCCAGGGTTTCTTACT
Homo-GM-CSF	AGCCCTGGGAGCATGTGAAT	GCAGCAGTGTCTCTACTCAGG
Homo-IL-1β	CAACAAGTGGTGTTCTCCATGTC	ACACGCAGGACAGGTACAGA
Homo-IL-6	CAATGAGGAGACTTGCCTGGT	GCAGGAACTGGATCAGGACT
Homo-IL-8	CTCTGTGTGAAGGTGCAGTTTT	GTTTTCCTTGGGGTCCAGACA
Homo-β-actin	GAAGAGCTACGAGCTGCCTGA	CAGACAGCACTGTGTTGGCG

### Effect of LBH589 on the *T. gondii* ultrastructure by transmission electron microscopy

The RPE cells were inoculated into a 75 cm culture flask and infected with tachyzoites at a ratio of 10:1 parasites/cells. LBH589 (750 nM) was added and the treatment was continued for additional 48 h. The cells were harvested and preserved in TEM fixative at least 24 h for agarose pre-embedding. After 2 h of post-fixation in 1% OsO_4_, the samples were dehydrated in a gradient at room temperature and then osmotically embedded for sectioning. The tissue sections were stained with 2% uranium acetate saturated alcohol solution and images were collected by transmission electron microscope (TEM, Hitachi, Tokyo, Japan).

### Animal model of ocular toxoplasmosis

C57BL/6 female mice (PengYue, Jinan, China), 6-8 weeks, were used for the ocular model of *T. gondii* infection. After the pupils of the anesthetized mice were dilated, the eyes were observed under a surgical microscope and a tiny incision was made 1 mm behind the limbus. Free tachyzoites (4000/2 μl) were injected through a Hamilton syringe into the vitreous, making sure that the tip does not damage the lens or retina (Smith et al., 2020). At 12 h after infection, the treatment group was injected with different doses of LBH589 (1, 2, 3 ng/μl) *via* intravitreal injection, and the infection group was injected with an equal volume of PBS. One week post injection, mouse eyes were subjected to follow-up experiments. All experiments involving mice in this study were approved by Laboratory Animal Ethical and Welfare Committee of Shandong University Cheeloo College of Medicine.

### Clinical investigations

One week after the intervention, the retinal structure of the anesthetized mice was assessed by spectral-domain optical coherence tomography (SD-OCT) using the RTVue XR Avanti devices (Optovue Inc, Fremont, CA, USA). The acquired image format was saved in a Tag Image File Format (TIFF) for analysis.

### Histopathology

Mouse eyeballs were taken immediately after euthanasia and fixed with eye fixative for 24 h. The tissue was then embedded in paraffin and cut into 4-μm-thick sections for hematoxylin and eosin (HE) staining. Sections were viewed for signs of pathology under a light microscope.

### Statistical analysis

Data in all experiments were analyzed and graphed using GraphPad Prism 8.0 unless otherwise stated. Each experiment was repeated independently three times. Data are presented as means ± SD. Student’s *t*-test was used to determine the statistical significance of the two-group comparison. Data results with *P* < 0.05 were indicated statistically significant.

## Results

### Toxicity of LBH589 on RPE cells

**Figure 1:**
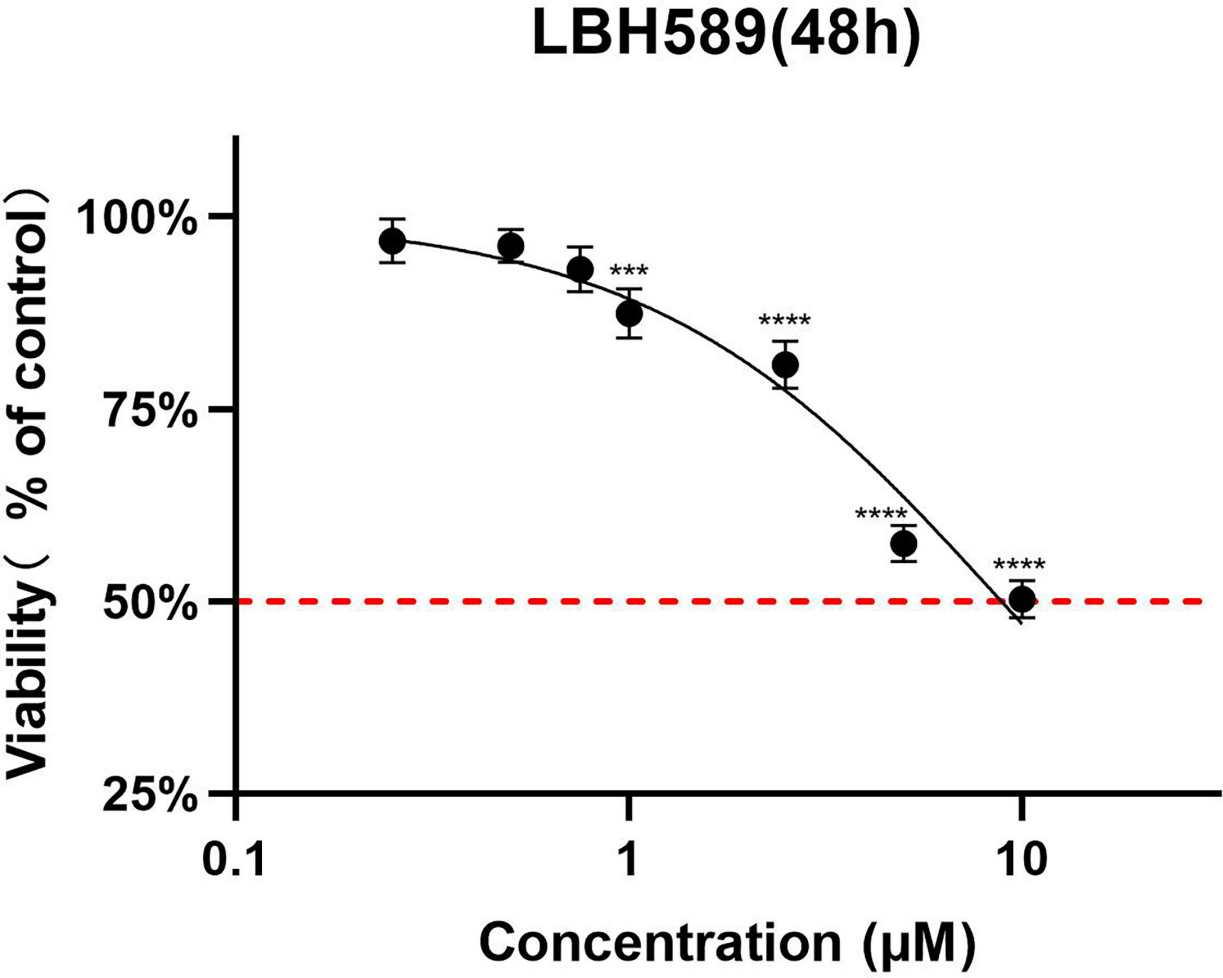
Cell toxicity assay of LBH589 on RPE cells for 48h of incubation. To determine a safe drug concentration *in vitro*, RPE cells were treated with different concentrations of drugs. RPE cells treated with DMEM were used as a control. Data are expressed as percent inhibition of cell viability relative to the control. ****P < 0.001, ***P < 0.0001 in comparison with the control. The experiment was repeated three times, with three wells of each sample.

To determine a safe drug concentration *in vitro*, we initiated experiments with RPE cells using different concentrations of the drug. The analysis of the results showed that the CC50 of the LBH589 on RPE cells was 8325.33 ± 407.18 nM for 48 h of treatment (**Table 2**). When the final concentration was less than 750 nM, LBH589 had no significant effect on cells viability (**Figure 1**).

**Table 2:** Selective Indexes for RPE cells after treatment with LBH589 for 48 h.

Compound	tachyzoite of RH	RPE cell	SI (Selective Index)
	IC50 (nM)	CC50 (nM)	(CC50/IC50)
LBH589	424.41 ± 48.07	8325.33 ± 407.18	19.62

### Antiproliferative effect of LBH589 against tachyzoites *in vitro*

**Figure 2:**
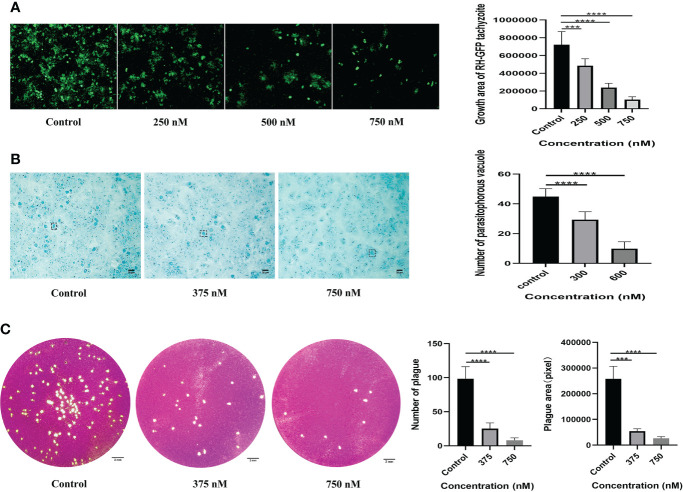
Assessing the effect of LBH589 on the proliferation and activity of *T. gondii*. Infected cells cultured with DMEM were used as negative controls. **(A)** The fluorescence area shows the proliferation of *T. gondii* after incubation with different concentrations of drugs for 48 h. **(B)** The number of intracellular parasitophorous vacuole in the treated and infected groups. **(C)** Comparing the overall growth of parasites in the infection group to that of the treatment group *via* plaque assay. ***P < 0.001, ****P < 0.0001, in comparison with the control. Scale bars: B = 20 μm; C= 2 mm. Each experiment was repeated three times, with three wells of each sample.

According to the experimental results, tachyzoites exhibited dose-dependent proliferation rate inhibition after LBH589 treatment for 48 h. 50% inhibiting concentration (IC50) of the parasite growth was 424.41 ± 48.07 nM. The drug concentration at 750 nM inhibited the proliferation rate by nearly 90% and showed no obvious toxic effect on cells (**Figures 1**, **2A**). Besides, the LBH589 selectivity index (CC50/IC50) was 19.62, which indicated the cellular safety of LBH589 in inhibiting the proliferation of *T. gondii* (**Table 2**). The Giemsa staining experiment also found that there were no obvious parasitophorous vacuoles in the cells under the drug concentration of 750 nM (**Figure 2B**). These data confirmed that strong inhibitory effect of LBH589 on *T. gondii* proliferation.

### Antiparasitic activity of LBH589 *in vitro*

To verify whether LBH589 affects parasite viability by plaque assay, we found that the size and number of plaques decreased after *T. gondii* pre-treatment within the safe concentrations of LBH589. As the drug concentration increased, the reduction of parasites was more significant compared to the control group. The number and plaque area of parasites pretreated with LBH589 (750 nM) were reduced by approximately 90% and 92%, respectively (**Figure 2C**).

### Expressions of GM-CSF, IL-1β, IL-6 and IL-8 genes in the *T. gondii*-infected and treated cells

**Figure 3:**
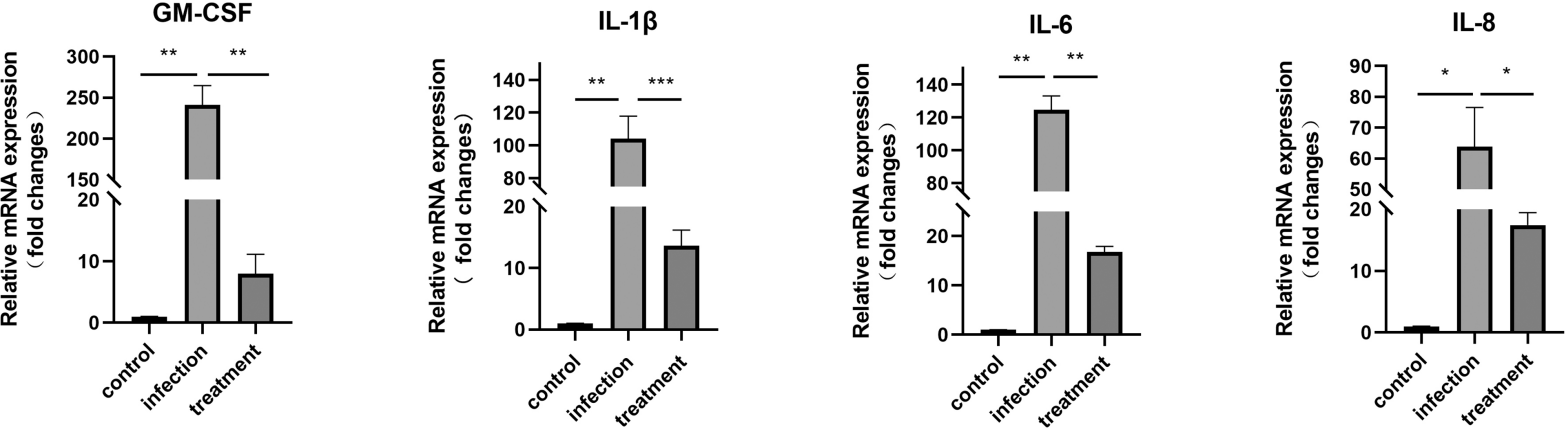
mRNA expression of GM-CSF, IL-1β, IL-6 and IL-8 in *T. gondii*-infected RPE cells was verified by qRT-PCR after 48 h of LBH589 treatment. Normal RPE cells served as the control. *P < 0.05, **P < 0.01, ***P < 0.001. Each experiment was repeated independently three times, with three wells of each sample.

The results revealed that compared with the control, the mRNA expression levels of GM-CSF (*P* < 0.01), IL-1β (*P* < 0.01), IL-6 (*P* < 0.01) and IL-8 (*P* < 0.05) were significantly increased in the *T. gondii*-infected group. When infected cells were treated with LBH589 at a concentration of 750 nM, levels of GM-CSF (30.19-fold, *P* < 0.01), IL-1β (7.64-fold, *P* < 0.001), IL-6 (16.78-fold, *P <*0.01) and IL-8 (3.68-fold, *P <*0.05) were decreased compared with the infected group (**Figure 3**).

### Ultrastructural changes in intracellular *T. gondii* tachyzoites

**Figure 4:**
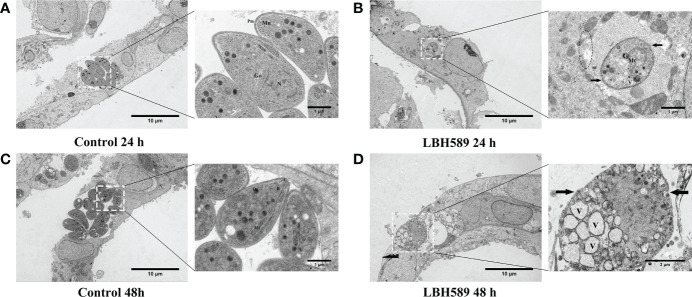
Ultrastructural alterations of tachyzoites internalized in RPE cells after LBH589 treatment at 750 nM by TEM. **(A, C)** Untreated parasites in RPE cells (set as the control) present normal morphology at both 24 and 48 h, the inset shows the typical ultrastructure, including the conoid (C), microneme (Mn), rhoptry (R), plasmalemma (Pm), dense granule (Dg), mitochondrion (Mt), golgi apparatus (G), and nucleus (N). **(B)** Exposure to LBH589 for 24 h induced unusual microstructural changes, such as mitochondrial swelling, vacuole (V), disruption of plasmalemma (black arrow) and vacuolar membranes (black circle). **(D)** After 48 h of incubation with LBH589, the parasites lost their biological features and the cytoplasmic structure was almost completely destroyed. Scale bars: A, B, C and D = 10 μm; A, B and C insets = 1 μm, D inset = 2 μm. The experiments were performed in triplicate, in three independent experiments.

After 24 h incubation with LBH589, the tachyzoites showed disorder of organizational structure. TEM findings showed mitochondrial swelling, vacuole formation, damaged plasmalemma and vacuolar membrane (**Figure 4B**). LBH589 treatment of the parasites for 48 h caused abundant empty vacuolization in cytoplasm. Besides, the cytoplasmic structure, plasmalemma and vacuolar membranes of tachyzoites had completely disappeared (**Figure 4D**). However, typical morphology and cytological features of tachyzoite were maintained completely incubated in the absence of LBH589 (**Figures 4A, C**).

### Retinal structure in OCT investigation

**Figure 5:**
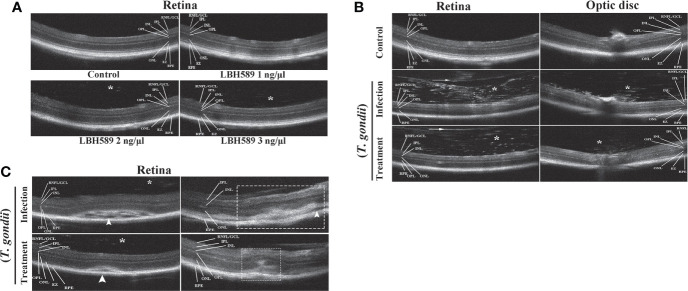
Clinical evaluation of vitreous cavity and retina structure after intravitreal injection of LBH589 by OCT. Normal mice were set as controls. **(A)** Scattered hyper-reflected signals (asterisk) are observed in the vitreous from intravitreal LBH589-injected (≥ 2 ng/1 μl) eyes. **(B, C)** Changes in the structure of retina and vitreous cavity of *T. gondii*-infected and LBH589-treated mice. The changes show dense hyper-reflective tiny dots (asterisk), posterior vitreous detachment (white arrow), serous retinal detachment (white arrowhead), blurred full thickness retinal, hyper-reflectivity foci and disorganized of retinal layers (white rectangle). Data are representative of results obtained from six mice in each group.

We determined whether intravitreal injection of LBH589 affects the intraocular conditions of mice by OCT examination. There were no significant changes on the retinal structure with 1ng of LBH589, but when the dose exceeded 2 ng injected into the eye, it caused varying degrees of vitreous opacity that was not observed in normal mice (**Figure 5A**). In OCT sets of the infected group, hyper-reflective tiny dots abutting the optic nerve head (papillitis) and floating in vitreous cavity above posterior hyaloid face (vitritis) were clearly observed (**Figure 5B**). In addition, the hyper-reflectivity foci accompanied by blurring of details in inner retina, disorganizing of retinal layers and serous retinal detachment were common. The degree of vitreous opacity and the size of the lesion of the subretinal fluid were significantly reduced in most animals treated with LBH589. Moreover, the retinal structure was more complete, the damage site was relatively limited and the surrounding structures of the lesion can be clearly distinguished (**Figures 5B, C**).

### Retinal structure in histology analysis

**Figure 6:**
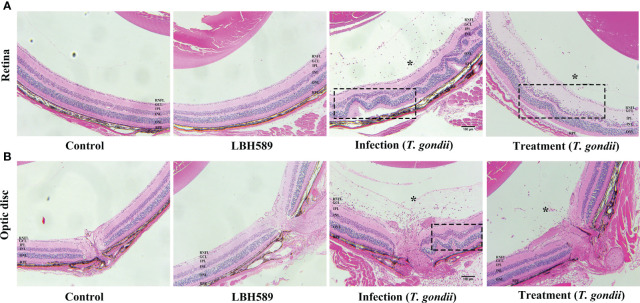
Histological changes of mice eyes. Compared with the control, histological retinal **(A)** and optic disc **(B)** changes in the eyes of LBH589-injected, tachyzoites-infected and LBH589-treated mice at 7 dpi such as inflammatory cell infiltrate (asterisk), retinal folding and destruction of retinal architecture (black rectangle). The retinal structure of normal mice injected with LBH589 alone was not significantly abnormal compared with the control. Scale bars: **(A, B)** = 100 μm. Data are representative of results obtained from six mice in each group.

Through histopathological examination, the intravitreal injection of tachyzoites caused a heavy inflammatory infiltrate involving peripapillary retina and the optic nerve head, leading to destruction of normal retinal structure. But the infiltration of inflammatory cells was alleviated and the retinal structure was relatively intact in the treatment group. Besides, there was no obvious abnormality in retinal structure for the intravitreal injection of LBH589 alone compared with the control group (**Figure 6**).

### Expressions of TNF-α, IL-1β and IL-6 genes in the untreated and treated mice

**Figure 7:**
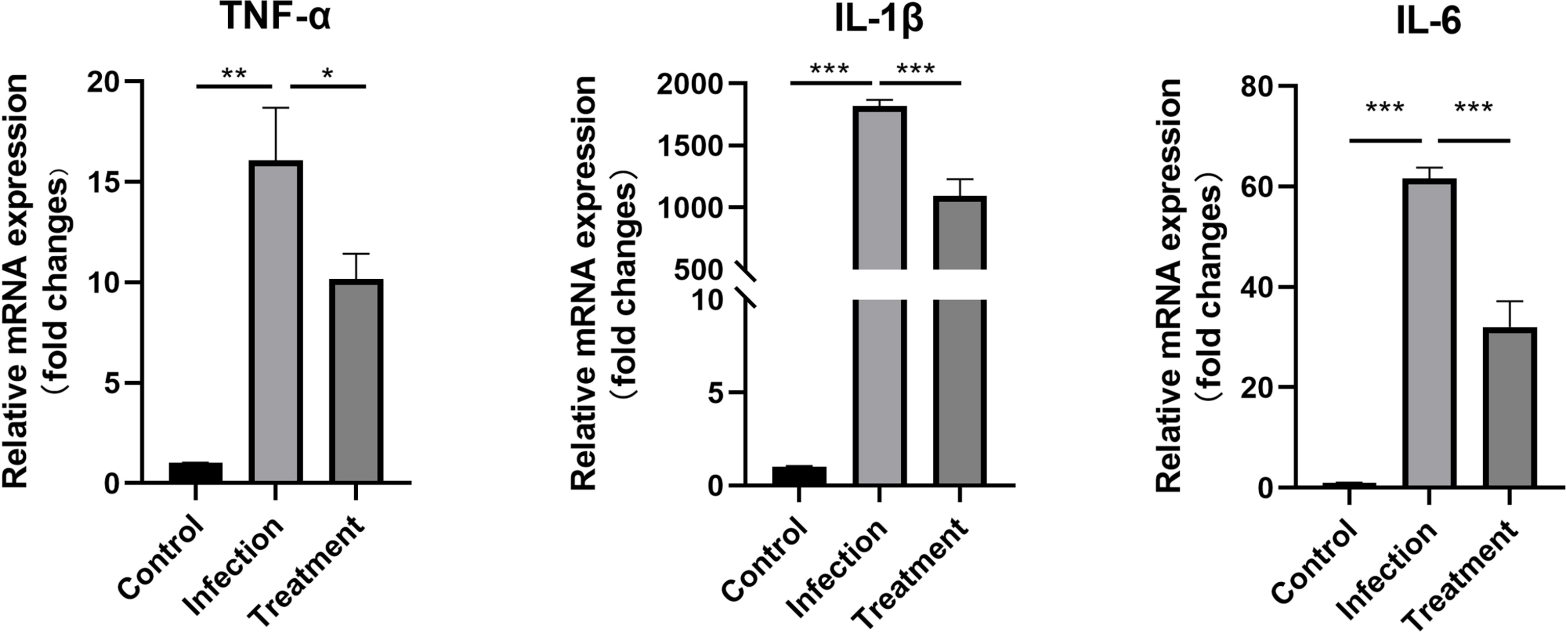
The differential mRNA expressions of TNF-α, IL-1β and IL-6 in the eyes of the control, tachyzoites-infected and LBH589-treated mice were measured by qRT-PCR. *P < 0.05, **P < 0.01, ***P < 0.001, the infection group versus the treatment group. The experiments were performed in triplicate, in three independent experiments.

Combined with previous literature reports, we selected several factors that were highly expressed in infected mice (Nagineni et al., 2000; Zhang et al., 2019). Compared with the control, the expression levels of TNF-α (*P* < 0.01), IL-1β (*P* < 0.001) and IL-6 (*P* < 0.001) were significantly increased at 1 week after infection. Compared with the untreated mice, the mRNA expression levels of pro-inflammatory cytokines TNF-α (1.58-fold, P < 0.05), IL-1β (1.67-fold, P < 0.001) and IL-6 (1.92-fold, P < 0.001) were significantly down-regulated in the treated group (**Figure 7**).

## Discussion

Ocular toxoplasmosis, as an infectious disease, often causes damage to the retinal structure during the acute phase, especially for immunocompromised patients. In addition, many patients are healthy adults with only ocular symptoms and no specific general symptoms prior to onset (Ozgonul and Besirli, 2017). The classic clinical treatment is the combined application of pyrimethamine and sulfadiazine (P-S). Trimethoprim and sulfamethoxazole (TMP-SMX) can also be used as alternative therapy and other optional drugs include clindamycin, azithromycin and atovaquone (Dunay et al., 2018). The major difficulty is that oral medication needs 4-6 weeks to control the disease, which may cause side effects such as anemia, myelosuppression and drug toxicity (Garweg and Pleyer, 2021). Besides, the existence of the blood-retinal barrier restricts the entry of most drugs into the eye.

During the development of anti-*T. gondii* drugs, HDAC inhibitors have been discovered as a potential drug (Jublot et al., 2022). The previous research has shown that anti-*T. gondii* effect of several HDAC inhibitors such as scriptaid, tubastatin A and suberoylanilide hydroxamic acid in HFF cells (Araujo-Silva et al., 2021). In our study, we have evaluated the anti-*T. gondii* and therapeutic effect of LBH589 on OT for the first time, which was mainly used in cutaneous T-cell lymphoma (CTCL), Hodgkin’s lymphoma as well as acute myeloid leukemia (AML) in preclinical research (Prince et al., 2009; Schroder et al., 2021; Chen et al., 2022). According to the pathogenesis of OT, retinal pigment epithelium has been identified to be the primary target for *T. gondii* infection within the eye (Smith et al., 2021). Unlike previous studies, we therefore selected RPE cells as host cell for subsequent vitro experiments. After 48 h of treatment, LBH589 was shown to inhibit proliferation of tachyzoites in a dose-dependent manner at concentrations ranging from 250 to 750 nM. According to the results of cytotoxicity assay, a selective index of 19.62 was achieved, and we have ensured the low toxicity of LBH589 to RPE cells in this concentration range. In addition, the activity of tachyzoites was significantly weakened after drug pretreatment *via* plaque assay. Therefore, these data preliminarily proved the inhibitory effect of LBH589 on *T. gondii in vitro*.

Five genes of HDAC class I/II (TgHDAC 1-5) have been found in *T. gondii*, which are responsible for the life cycle (Vanagas et al., 2012). In previous studies, it was found that HDAC inhibitors affect the cell division of *T. gondii* by regulating the acetylation state of histones H3 and H4, which cause damage to cell morphology and microstructure such as acroplasts, mitochondria and nucleus (Mouveaux et al., 2022). Moreover, HDAC inhibitors are capable of causing alterations in cytoskeletal protein such as a decrease in the α-tubulin amount, leading to the formation of masses of damaged parasites (Araujo-Silva et al., 2021). Our transmission electron microscopy also showed a severe damage on parasite pellicle and intracellular organelles such as the presence of nuclear fragmentation, mitochondrion swollen, plasma membrane rupture, a loss of crescent shape and ultimately complete destruction of the structure after treatment. Combined with the results of above experiment, LBH589 may have the ability to cause damage to the cellular structure of parasite by diffusing through the host cell plasma membrane and the parasitophorous vacuole.

In addition, during *T. gondii* infection, RPE cells were proved to enhance the release of proinflammatory cytokines, thereby inducing neutrophils to produce ROS, TNF-α and IL-1β, which further aggravated the tissue damage (Ashander et al., 2019; Zhang et al., 2019; Raouf-Rahmati et al., 2021). Our study has also revealed that after infection of RPE cells with parasites, mRNA expression of GM-CSF, IL-1β, IL-6 and IL-8 showed a statistical increase. 48 h after treatment, infected RPE cells significantly reduced the mRNA expression of four pro-inflammatory factors. The reason for this phenomenon may be that LBH589 inhibits the proliferation of tachyzoites and thus weakens the stimulation of cells.

In the animal model, we induced acute ocular toxoplasmosis by intraocular injection of tachyzoites. It has been reported that the advantage of this method is that it can breach ocular immune privilege and rapidly induces vitritis and retinitis before the onset of systemic disease in mice (Dunay et al., 2018; Smith et al., 2020). In terms of treatment, the general principle of the therapy is to inhibit parasites proliferation, immediately control the inflammatory response, and mitigate tissue damage to protect retina during acute infection (Casoy et al., 2020). Intravitreal injection can limit systemic side effects, increase intraocular drug concentration and enhance the treatment effect in a short time to control the disease, receiving a lot of attention in the research of infectious diseases (Ozgonul and Besirli, 2017). Prospective randomized trial of intravitreal injection of clindamycin has been found to have a better therapeutic effect than oral administration (Baharivand et al., 2013). Therefore, we also selected this method to evaluate the therapeutic effect of LBH589 instead of intraperitoneal injection or oral gavage. In order to minimize the secondary damage to the mouse’s eyes and set aside time for *T. gondii* infection, mice were injected with LBH589 into the eyes 12 h after infection with *T. gondii*, and performed clinical evaluation on the 7th day when the ocular symptoms of the mice were most obvious (LaGrow et al., 2017; Lafreniere et al., 2019; Kishimoto et al., 2019; Smith et al., 2020). Here, we first adopted the ophthalmic imaging diagnostic technology, OCT examination, which can directly non-invasive assessment of intraocular lesion in living mice (Cunningham et al., 2022). In the infection group, most of the mice developed severe vitreous, subretinal fluid, posterior vitreous detachment and disorder of retinal structure (Chen et al., 2016; Brandao-de-Resende et al., 2020; Ksiaa et al., 2022), while the symptoms were relatively mild with LBH589 treatment. In addition, we set a drug dose gradient of 1 to 3 ng/μl based on the previous study about pharmacokinetics of LBH589 to avoid the toxicity of drugs to the retina (Van Veggel et al., 2018; Karol et al., 2020; Homan et al., 2021). Though the OCT results found that when the drug concentration was greater than 1 ng, vitreous opacity occurred in the eyes of mice, which was easily confused with the symptoms caused by infection, but the retinal results were not significantly abnormal. At the drug concentration of 1ng, the result showed no obvious difference compared with the control. The reason may be caused by fine drug particles or effect of drug solubility.

However, acute ocular toxoplasma usually causes vitreous opacity or complicated cataract, leading to opacity of the refractive medium, which make it difficult for OCT examination to obtain high-quality images. Subsequently, histopathological studies also showed that the inflammatory cell infiltration and the damage to retinal architecture were drastically reduced upon treatment with LBH589. Retinal structure of uninfected mice injected with LBH589 alone showed no obvious abnormality. Combined with OCT examination and histopathology, we infer that LBH589 can play a certain role in the treatment of ocular toxoplasmosis *in vivo*.

Moreover, one of the reasons for the destruction of tissue structure is the excessive production of inflammatory cytokines (Zhang et al., 2019; Smith et al., 2021). HDAC inhibitors vorinostat, butyrate and tubastatin A showed promising therapeutic potential in the treatment of inflammatory diseases such as rheumatoid arthritis, asthma, contact hypersensitivity and inflammatory bowel diseases, due to their ability to regulate inflammatory cells and cytokines through several G protein–coupled receptors (GPCRs) (Ran and Zhou, 2019; Li et al., 2021; Ni et al., 2021). We found that the mRNA expression levels of TNF-α, IL-1β and IL-6 were significantly increased after 7 days of infection, while the expression of the three inflammatory factors was decreased upon intraocular injection of LBH589. Combined with cell experiments, we infer that LBH589 has a certain anti-inflammatory ability as well as the ability to inhibit the proliferation of *T. gondii*. Though our data can prove that LBH589 has potential therapeutic effect on ocular toxoplasmosis, pharmacokinetics and mechanism of action of the LBH589 in the eye need to be studied further.

In summary, our research demonstrated that LBH589 can effectively inhibit the proliferation and activity of *T. gondii* in RPE cells. And the microstructure of *T. gondii* was significantly damaged, we speculated that LBH589 should have the ability to penetrate host cell plasma membrane and directly damage *T. gondii*. *In vivo*, we established a mouse model of acute OT, and combined with OCT examination and pathological sections to evaluate the therapeutic effect of intravitreal injection of LBH589. In addition, LBH589 treatment can also reduce mRNA expression levels of inflammatory cytokines. However, pharmacokinetics and the underlying mechanism of LBH589 still warrants further exploration.

## Data availability statement

The original contributions presented in the study are included in the article/supplementary material. Further inquiries can be directed to the corresponding authors.

## Ethics statement

The animal study was reviewed and approved by Laboratory Animal Ethical and Welfare Committee of Shandong University Cheeloo College of Medicine.

## Author contributions

YQ revised the manuscript. HC directed the project. YZ supervised the experiments and wrote the manuscript. QZ and HL conducted the experiments. All authors contributed to the article and approved the submitted version.

## Acknowledgments

We thank Dr. Bing Han and Dr. Huaiyu Zhou for providing the *T. gondii* strain and HFF cells and Dr. Chunxue Zhou for the experimental operation guidance.

## Conflict of interest

The authors declare that the research was conducted in the absence of any commercial or financial relationships that could be construed as a potential conflict of interest.

## Publisher’s note

All claims expressed in this article are solely those of the authors and do not necessarily represent those of their affiliated organizations, or those of the publisher, the editors and the reviewers. Any product that may be evaluated in this article, or claim that may be made by its manufacturer, is not guaranteed or endorsed by the publisher.
